# Reduction of AUF1-mediated follistatin mRNA decay during glucose starvation protects cells from apoptosis

**DOI:** 10.1093/nar/gku778

**Published:** 2014-08-26

**Authors:** Xiangwei Gao, Haojie Dong, Chen Lin, Jinghao Sheng, Fan Zhang, Jinfeng Su, Zhengping Xu

**Affiliations:** 1 Institute of Environmental Medicine, Zhejiang University School of Medicine, 866 Yuhangtang Road, Hangzhou 310058, China; 2 Program in Molecular Cell Biology, Zhejiang University School of Medicine, 866 Yuhangtang Road, Hangzhou 310058, China

## Abstract

Follistatin (FST) performs several vital functions in the cells, including protection from apoptosis during stress. The expression of FST is up-regulated in response to glucose deprivation by an unknown mechanism. We herein showed that the induction of FST by glucose deprivation was due to an increase in the half-life of its mRNA. We further identified an AU-rich element (ARE) in the 3′UTR of FST mRNA that mediated its decay. The expression of FST was elevated after knocking down AUF1 and reduced when AUF1 was further expressed. *In vitro* binding assays and RNA pull-down assays revealed that AUF1 interacted with FST mRNA directly via its ARE. During glucose deprivation, a majority of AUF1 shuttled from cytoplasm to nucleus, resulting in dissociation of AUF1 from FST mRNA and thus stabilization of FST mRNA. Finally, knockdown of AUF1 decreased whereas overexpression of AUF1 increased glucose deprivation-induced apoptosis. The apoptosis promoting effect of AUF1 was eliminated in FST expressing cells. Collectively, this study provided evidence that AUF1 is a negative regulator of FST expression and participates in the regulation of cell survival under glucose deprivation.

## INTRODUCTION

Follistatin (FST) was originally identified from follicular fluid based on its ability to suppress follicle-stimulating hormone secretion (1,2). The protein was later found to be expressed in nearly all tissues of higher animals and participate in a variety of processes such as cell growth, development, differentiation and secretion (3). Recently, several reports have shown that FST is also involved in tumor progression processes including angiogenesis (4), metastasis (5) and cell apoptosis (6). Most of FST functions have been attributed to its ability to extracellularly bind and inactivate transforming growth factor (TGF)-β-like molecules including activin, bone morphogenetic proteins (BMPs) and myostatin (7–9). There are two different transcripts, *FST317* and *FST344*, resulting from alternative mRNA splicing. During translation, the 29 amino acids secretory signal peptide in the protein precursors is cleaved, leading to formation of two mature isoforms. The larger isoform (FST-315) differs from the smaller one (FST-288) in having a COOH-terminal extension (10). Although the distributions of these two isoforms in human body are different, both of them can neutralize TGF-β family members.

Accumulating evidence suggests that FST plays diverse roles via mechanisms unrelated to its activin or BMP inhibitory activity. For instance, nuclear localization of FST was detected in both cultured cells (11) and breast cancer tissues (12). Our previous report has shown that the nuclear FST plays a protective role during cellular energy stress (13). Under glucose deprivation, the expression level of FST is up-regulated and the protein is translocated to the nucleoli in HeLa cells. Localization of FST to the nucleolus attenuated ribosomal RNA (rRNA) synthesis and ribosome biogenesis, which in turn delayed cell apoptosis (13). These data suggested that FST is important for cellular energy homeostasis and cell survival under glucose deprivation. However, the mechanism of FST regulation by glucose depletion remains to be elucidated.

Regulation of mRNA decay rates is an important mechanism by which gene expression is controlled at the post-transcriptional level (14). Decay rates are directed via *cis*-acting sequence elements contained within each mRNA. Adenylate/uridylate-rich elements (AREs) are the best characterized regulatory elements present in the 3′-untranslated region (3′UTR) of certain mRNAs that have been implicated in post-transcriptional gene regulation by mediating rapid degradation of target mRNAs (15). Typical AREs contain one or more AUUUA elements within an AU-rich region (16). These RNA sequences serve as binding sites for RNA-binding proteins that may positively or negatively impact the mRNA degradation process. Several ARE-binding proteins (ARE-BPs), such as the AU-rich element-binding protein 1 (AUF1) family, human antigen R (HuR) family and the tristetraprolin (TTP) family have been identified to exert a defined role in ARE-mRNA turnover (15). The interaction between *cis* elements in the transcripts and *trans*-acting factors permits precise control of the mRNA level and, subsequently, the protein level.

AUF1, also known as heterogeneous nuclear ribonucleoprotein D (hnRNPD), is the first ARE-BP to be isolated and for which a role in controlling mRNA stability has been reported (17). AUF1 comprises four proteins that arise from alternative splicing (p37, p40, p42 and p45) and it shuttles between the nucleus and the cytoplasm (18). In the nucleus, the protein regulates multiple cellular processes such as telomere maintenance (19,20), transcriptional activation (20), as well as pre-mRNA processing (21). In the cytoplasm, AUF1 binds to several mRNAs, including c-myc, c-fos, Cyclin D1, IL-3, etc., and promotes mRNA decay. On the other hand, AUF1-binding has also been associated with increased mRNA stability, such as PTH, TNF-α (15). The effect of AUF1 on mRNA stability might also be cell-type specific: destabilizing in one cell type (22) while stabilizing in another cell type (23), suggesting the complexity of AUF1-regulated mRNA turnover.

In this study, we showed that the ARE-BP AUF1 interacts with the 3′UTR of FST mRNA and lowers the stability of the FST mRNA. Glucose limitation triggered dissociation of AUF1 from the FST mRNA, leading to significant increase in its stability. We propose that the post-transcriptional stabilization of FST mRNA contributes to elevated FST levels in cells responding to energy deprivation.

## MATERIALS AND METHODS

### Cell culture and treatments

HeLa human cervical carcinoma cells were obtained from ATCC (American Type Culture Collection) and maintained in Dulbecco's modified Eagle's medium (DMEM) containing 25 mM glucose (Invitrogen, Carlsbad, CA, USA) supplemented with 10% fetal bovine serum (Thermo Fisher Scientific, Waltham, MA, USA). Cells were maintained at 37°C in an atmosphere containing 5% CO_2_ and 100% humidity. For glucose deprivation treatment, cells were washed with phosphate buffered saline (PBS) and incubated with glucose-free DMEM (Invitrogen) for indicated time.

### RNA purification and reverse transcription reaction

Total RNA was isolated with Trizol reagent (Invitrogen) following the manufacturer's protocol. 0.5 μg of total RNA was reverse transcribed using random hexamers and the High Capacity cDNA Reverse Transcription Kit (Life Technologies, Grand Island, NY, USA).

### qPCR measurement

Real-time quantitative PCR analysis was performed in 10-μl reactions using the ABI7900 (Applied Biosystems) and SYBR GREEN PCR Master Mix (Applied Biosystems). Primers 5′-TTGCGTTACACCCTTTCTTG-3′ and 5′-CACCTTCACCGTTCCAGTTT-3′ were used for β-actin gene amplification. Primers 5′-ACGGATTTGGTCGTATTGGG-3′ and 5′-CGCTCCTGGAAGATGGTGAT-3′ were used for GAPDH gene amplification. Primers 5′-CGACGACCCATTCGAACGTCT-3′ and 5′-CTCTCCGGAATCGAACCCTGA-3′ were used for 18S rRNA gene amplification. Primers 5′-TCTAAGGAAGTCGGGGAAGC-3′ and 5′-CCCTCGGGTGTAATCAGAAT-3′ were used for Luc gene amplification. Primers 5′-GGGAACTGCTGGCTCC-3′ and 5′-TTTACAGGGGATGCAG-3′ were used for total FST gene amplification. Primers 5′-CTGTTGTTGTCCGTATTT-3′ and 5′-GTAGTCCTGGTCTTCATCT-3′ were used for FST317 amplification. Primers 5′-GATCTTGCAACTCCATTTCG-3′ and 5′-AGGCTATGTGAACACTGAAC-3′ were used for FST344 amplification. Expressions of FST gene and its isoforms were normalized relative to β-actin or 18S rRNA endogenous control using the 2^−ΔΔCT^ method (24).

### Plasmid construction

The 3′UTRs of FST344 and its deletion mutants were amplified by RT-PCR reaction (Supplementary Table S1) and cloned into pGL3-control vector (Promega, Madison, WI, USA) or pcDNA3.1 (Invitrogen). The sequences of all primers are listed in Supplementary Table S2.

### Luciferase assay

HeLa cells were transfected with each firefly luciferase construct or together with siRNA. pRL-TK was co-transfected as the internal control. Cells were lysed and luciferase activity was measured by the Dual-Luciferase assay system (Promega) 48 h after transfection. The firefly luciferase activity was normalized to Renilla luciferase.

### RNA interference

For knockdown experiments, HeLa cells were transiently transfected with 40 nM of the chemically synthesized siRNAs targeting AUF1 or with the negative control siRNA using Lipofectamine2000 (Invitrogen) according to the manufacturer's recommendations. Cells were harvested or treated for further experiments 48 h after transfection. siRNA sequences used in the present study are designed as follows: AUF1 siRNA, forward, GGGUCCCUCUGAAGUUUAATT, reverse, UUAAACUUCAGAGGGACCCTT; negative control siRNA, forward, UUCUCCGAACGUGUCACGUTT, reverse, ACGUGACACGUUCGGAGAATT. siRNAs were synthesized by GenePhama company (Shanghai, China).

### Protein extraction and fractionation

The whole-cell lysate was extracted with RIPA (Radioimmunoprecipitation assay buffer) buffer (100 mM Tris at pH 8.0, 1% Triton X-100, 100 mM NaCl, 0.5 mM ethylenediaminetetraacetic acid (EDTA)) with freshly added complete protease inhibitor cocktail (Roche Applied Science, Indianapolis, IN, USA). To detect the subcellular distribution of AUF1, cytoplasmic and nuclear fractions were prepared according to the previously reported method (25) with modifications. Briefly, the cells were washed with PBS and resuspended in hypotonic buffer (10 mM HEPES (4-(2-Hydroxyethyl)piperazine-1-ethanesulfonic acid) at pH 8.0, 10 mM KCl, 3 mM MgCl_2_, 0.5 mM DTT (Dithiothreitol) and protease inhibitors). The suspension was incubated on ice for 10 min before adding Triton X-100 to a final concentration of 0.3% to allow a sufficient breakage of the cytoplasmic membranes. After centrifugation at 500 g for 5 min at 4°C, the resultant supernatant was used as the cytoplasmic fraction. The nuclear pellet was washed twice with hypotonic buffer and reconstituted in RIPA buffer. The resultant supernatant was used as the nuclear lysate.

### Immunoblotting analysis

Proteins were quantified using a modified Bradford protocol (BioRad Laboratories, Hercules, CA, USA) and applied to immunoblotting analysis as described previously (26). Equal amounts of proteins were subjected to sodium dodecyl sulfate-polyacrylamide gel electrophoresis (SDS-PAGE) electrophoresis and transferred to nitrocellulose membrane (Whatman, Clifton, NJ, USA). Membrane was blocked with 3% bovine serum albumin (BSA) TBS-T buffer (20 mM Tris–HCl, pH 8.0, 150 mM NaCl, 0.05% Tween-20), probed with antibodies targeting to FST (Santa Cruz Biotechnology, Santa Cruz, CA, USA), AUF1 (Millipore Corporation, Billerica, MA, USA), GAPDH (Cell Signaling Technology, Beverly, MA, USA), Lamin (Santa Cruz Biotechnology) or cleaved PARP (Cell Signaling Technology), incubated with horseradish-conjugated secondary antibodies, detected with the SuperSignal West Pico chemiluminescence substrate (Thermo Fisher Scientific), and finally exposed to an X-ray film.

### Immunofluorescence detection

Cells grown on glass coverslips were rinsed with PBS and fixed in 4% formaldehyde in PBS for 15 min. After rinsing twice with PBS, the cells were permeabilized in 0.2% Triton X-100 in PBS, and blocked with goat serum for 1 h at room temperature. The cells were then incubated with anti-AUF1 antibody (R&D Systems, Minneapolis, MN, USA) overnight, stained with the appropriate fluorescein isothiocyanate (FITC)-conjugated secondary antibody in PBS for 1 h and mounted on microscope slides. Images were obtained with a Zeiss immunofluorescence microscope (Carl Zeiss MicroImaging, Thornwood, NY, USA).

### Immunoprecipitation assays

Immunoprecipitation of RNP (Ribonucleoprotein) complexes was performed as described (27). HeLa cells were washed with PBS and irradiated with 650 J/m^2^ Ultraviolet-C for the crosslinking of RNA–protein complexes in intact cells. Cells were then lysed with lysis buffer (PBS containing 1% Empigen BB). After centrifugation, the supernatant was incubated with IgG or AUF1 antibody for overnight. Protein A agarose beads were added to the samples and incubated for 1 h. Beads were then washed three times with lysis buffer and once with NT2 buffer (50 mM Tris–HCl pH 7.4, 150 mM NaCl, 1 mM MgCl_2_ and 0.5% Nonidet P-40). For the analysis of RNA in the immunoprecipitation material, beads were incubated with NT2 buffer containing 20 U RNase-free DNase I for 15 min at 30°C, washed with NT2 buffer, and further incubated in NT2 buffer containing 0.1% SDS and 0.5 mg/ml proteinase K at 55°C for 15 min. RNA was extracted and subjected to RT-qPCR analysis. Alternatively, bound proteins were boiled and examined by western blotting.

### *In vitro* biotin-labeled RNA pull down

The 3′UTR of FST317 and its mutants were cloned to pcDNA3.1 and mRNA was synthesized through *in vitro* transcription using mMESSAGE mMACHINE T7 ULTRA Kit (Ambion). The RNA fragments were biotinylated using RNA 3′ End Biotinylation Kit (Thermo Fisher Scientific).

Biotin pull-down assays were carried out by incubating cytoplasmic fractions with biotinylated transcripts for 1 h at room temperature. Complexes were isolated with paramagnetic streptavidin-conjugated Dynabeads (Dynal, Oslo, Norway), and bound proteins in the pull-down material were analyzed by western blotting using AUF1 antibody.

### *In vitro* RNA binding assay

GST fusion proteins were expressed in *Escherichia coli* BL21 and purified by affinity chromatography on GSTrap FF columns (GE Healthcare, Little Chalfont, Buckinghamshire, UK) following the manufacturer's protocol. FST 3′UTR RNA was transcribed using mMESSAGE mMACHINE T7 ULTRA Kit (Ambion).

Binding of GST-AUF1 isoforms to FST 3′UTR were measured by a gel-shift assay as described previously (28). In brief, 1 μg of FST 3′UTR RNA was incubated with purified proteins in the *in vitro* binding buffer (20 mM HEPES pH 8.0, 30 mM NH_4_Cl, 100 mM KCl, 0.5 mM MgCl_2_, 1 mM DTT, 4% glycerol, 0.1% Nonidet P-40, 0.05 μg/μl of BSA, 0.4 unit/μl of RNase inhibitor) at room temperature for 1 h. Samples were separated by the 1.2% agarose gel and stained with ethidium bromide. The shift of the bound RNAs were visualized under ultraviolet (UV) light.

### Apoptosis assays

Both floating (dead) and attached cells were harvested and applied to different assays for apoptosis detection. Cells were incubated with 5 μl of annexin V-FITC (Biovision, Mountain View, CA, USA) and 10 μl of protease inhibitor for 10 min and analyzed by flow cytometry (Beckman Coulter, Brea, CA, USA). Annexin V-FITC staining was detected in the FL1 channel, whereas PI staining was monitored in the FL3 channel. Alternatively, total proteins were extracted and cleaved poly ADP ribose polymerase (PARP) was detected by immunoblotting.

## RESULTS

### Glucose deprivation increases the stability of FST mRNA

**Figure 1.**
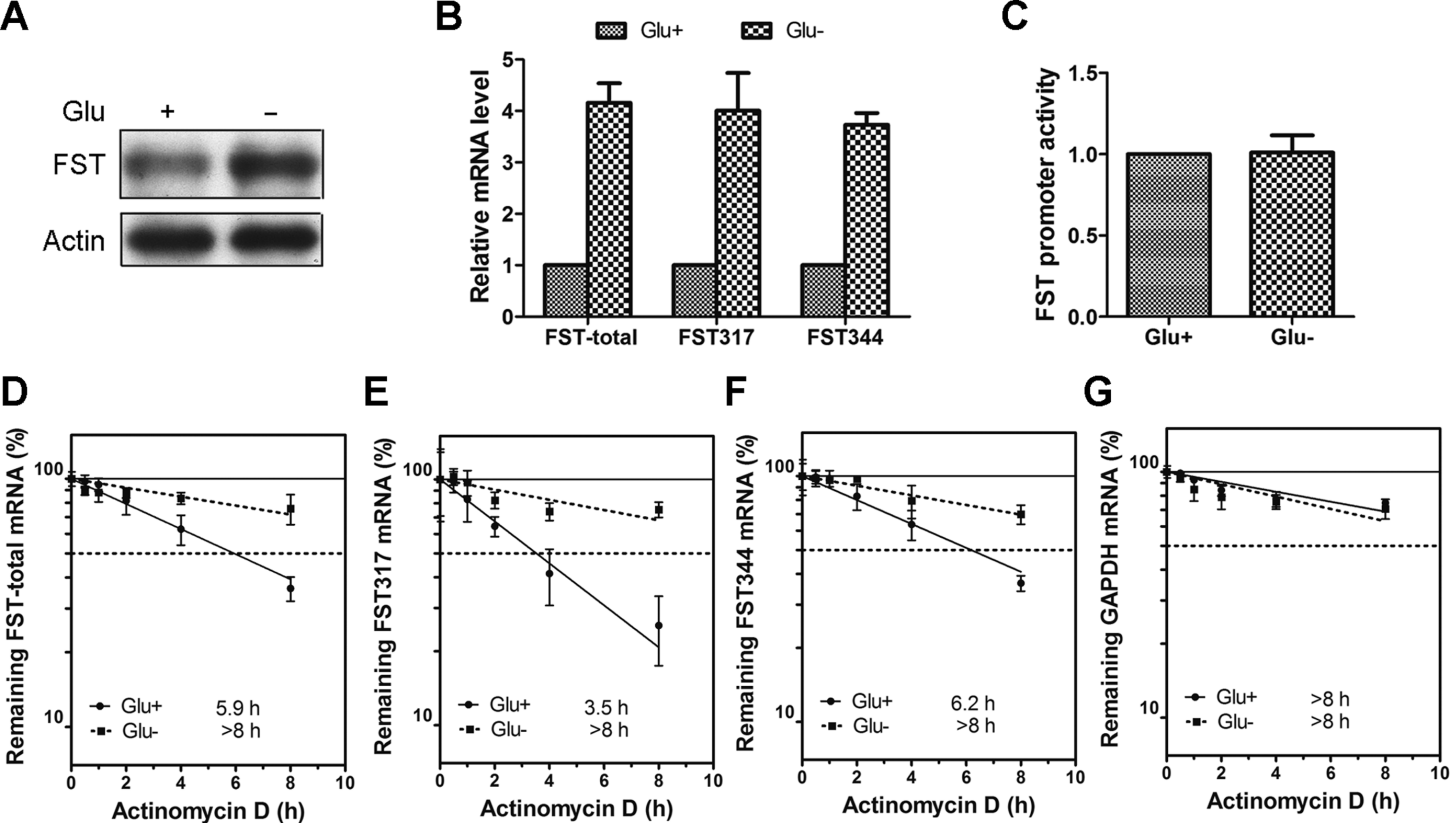
Glucose deprivation increases the stability of FST mRNA. (**A**) HeLa cells were incubated with (Glu+) or without (Glu−) glucose for 24 h and the protein level of FST was detected by immunoblotting. (**B**) HeLa cells were incubated with or without glucose and the mRNA levels of total FST, FST317 and FST344 were measured with real-time qPCR. Data shown are mean ± SD of three independent experiments. (**C**) HeLa cells transfected with FST promoter construct were incubated with or without glucose and luciferase activity was detected. Data shown are mean ± SD of three independent experiments. The mRNA levels of total FST (**D**), FST317 (**E**), FST344 (**F**) or GAPDH (**G**) in cells cultured with or without glucose were measured following treatment with actinomycin D for 0.5, 1, 2, 4 and 8 h. All the mRNA levels were normalized to 18S rRNA level. The half-life of each mRNA is defined as the time needed to reach 50% of its original abundance at time 0 h (dashed line). Data shown are mean ± SD of three independent experiments.

We recently showed that glucose deprivation in HeLa cells increases the expression of FST protein, which thereby delayed cell apoptosis (13). In keeping with the study, glucose deprivation for 24 h increased the protein (Figure 1A) and mRNA level (Figure 1B) of FST. FST mRNA has two transcripts, *FST317* and *FST344*, derived from alternative splicing (10). We therefore designed primers to detect the expression level of each transcript. RT-qPCR data showed that glucose deprivation increased the mRNA level of both transcripts (Figure 1B). The higher level of FST mRNA under glucose deprivation may result from either transcriptional activation or post-transcriptional regulation. To distinguish between these two levels of regulation, we first detected changes in FST gene promoter activity using a luciferase reporter (29). Data showed that the transcriptional activity of FST promoter region was not significantly induced under glucose deprivation (Figure 1C). Next, we examined the stability of FST mRNA. Actinomycin D was used to block *de novo* RNA synthesis, and then the persistence of the existing FST mRNA was measured by quantitative RT-qPCR at 1, 2, 4 and 8 h. Our results revealed that glucose deprivation led to substantial stabilization of the FST mRNA. The half-life of total FST mRNA is longer in glucose-starved than control cells (Figure 1D, >8 h versus 5.9 h). The half-life of both *FST317* (>8 h versus 3.5 h) and *FST344* (>8 h versus 6.2 h) increased in the absence of glucose (Figure 1E and F). These results suggested that glucose deprivation-triggered FST mRNA up-regulation is due to mRNA stabilization, not transcriptional activation.

### The 3′UTR of FST mRNA bears a destabilization element responsive to glucose deprivation

**Figure 2.**
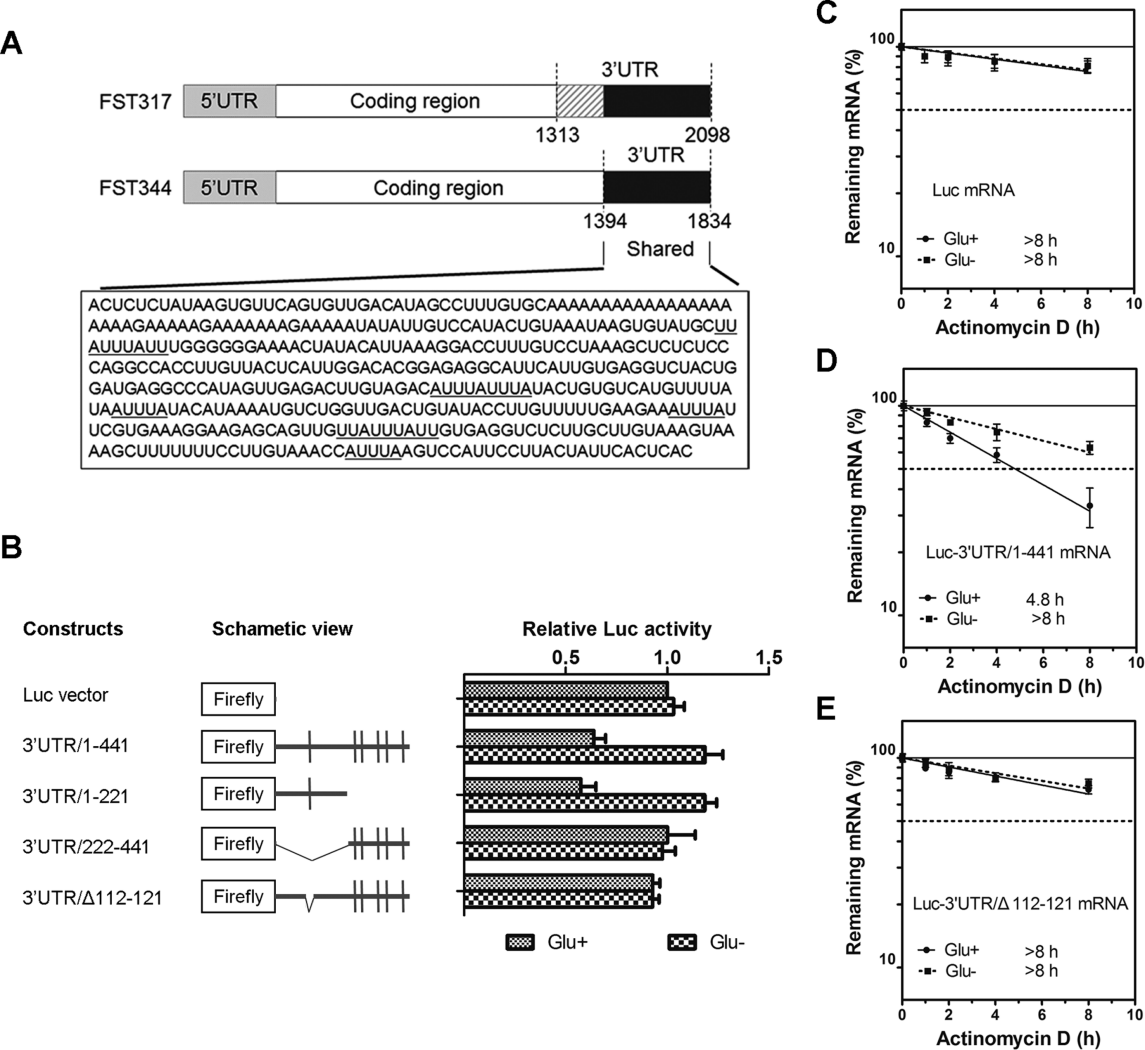
The 3′UTR of FST mRNA bears a destabilization element responsive to glucose deprivation. (**A**) Schematic illustration of two transcripts of FST gene. The sequence corresponding to the 3′UTR of FST344 is depicted. The potential ARE sequence (AUUUA) is underlined. (**B**) Luciferase reporter activities of deletion mutants of FST 3′UTR under normal and glucose-deprivation conditions. The left panel is the schematic views of the deletion constructs. The right graphs are luciferase activities of these constructs normalized to pRL-TK. Data shown are mean ± SD of three independent experiments. Luc gene (**C**), Luc gene fused with FST 3′UTR (**D**) or ARE-deleted mutant (**E**) was transfected into HeLa cells. Twenty-four hours after transfection, cells were cultured with or without glucose for 12 h and the mRNA levels were detected at indicated times following actinomycin D treatment. Data shown are mean ± SD of three independent experiments.

AU-rich element (ARE) sequences, which are present in the 3′UTR of certain mRNAs, function post-transcriptionally to regulate the mRNA stability (15,16). By analyzing the two transcripts of FST mRNA, we found that *FST344* has a shorter 3′UTR, which is also a part of the longer 3′UTR of *FST317*. Further analysis showed that this shared region is AU-rich with 33% of adenine and 34% of uracil and contains six potential AREs (AUUUA) (Figure 2A). To examine whether this region can regulate mRNA stability, we fused it after the stop codon of luciferase (Luc) gene and measured the luciferase activity. Insertion of the shared region reduced luciferase activity by ∼50% (Figure 2B), implying that it has a destabilizing role. To identify which ARE is responsible for this destabilization, a series of truncated 3′UTR fragments were generated. The mutants containing the first ARE (ARE1) decreased luciferase activity, while mutants lacking ARE1 did not have any effect (Figure 2B), indicating that ARE1 is the functional ARE. Next, we explored the response of ARE1 to glucose deprivation. Data showed that Luc activities of reporters containing ARE1 increased significantly (∼2-fold) after glucose deprivation (Figure 2B). To further confirm the destabilizing role of ARE1, we detected the mRNA stability of each Luc construct. The Luc mRNA itself was rather stable (>8 h), regardless of glucose deprivation or not (Figure 2C). Addition of full-length 3′UTR decreased Luc mRNA half-life (4.8 h), while glucose deprivation increased its half-life (>8 h) (Figure 2D). ARE1-deleted mutant did not affect the half-life as Luc (Figure 2E, >8 h). These findings demonstrated that the ARE1 is a negative regulatory element that responds to glucose deprivation. Our data also indicated that the increased expression of FST during glucose deprivation relies on this ARE.

### AUF1 promotes FST mRNA decay

**Figure 3.**
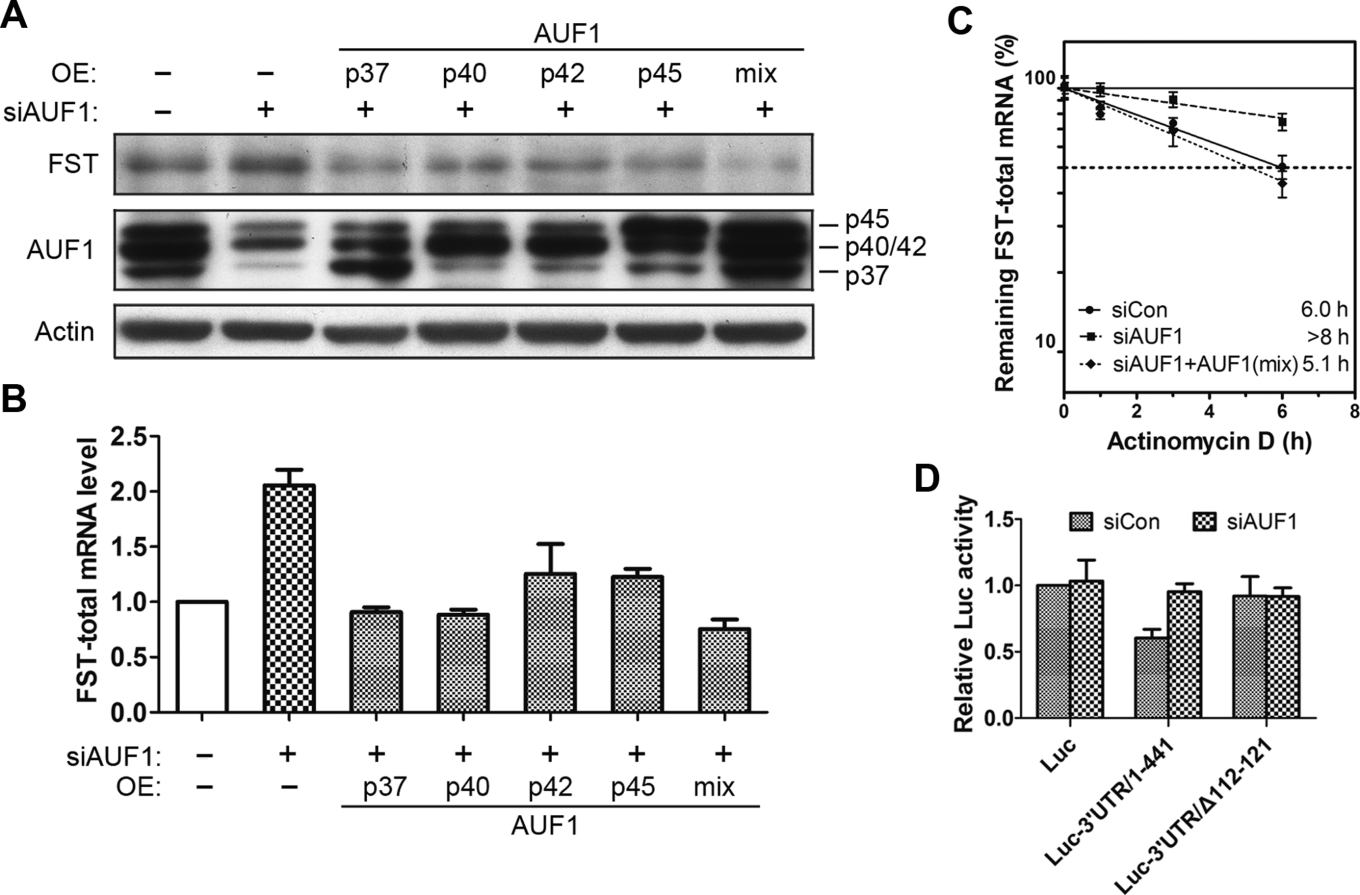
AUF1 promotes FST mRNA decay. (**A**, **B**) HeLa cells were transfected with siRNA targeting AUF1 together with or without plasmids expressing AUF1 isoforms. The protein level (A) and mRNA level (B) of FST were detected. (**C**) FST mRNA half-life was detected in cells transfected with siRNA targeting AUF1 together with or without plasmids expressing AUF1 isoforms mixture. (**D**) HeLa cells were transfected with siRNA targeting AUF1 together with or without plasmids expressing AUF1 isoforms. FST mRNA levels were detected at indicated times following actinomycin D treatment. Data shown are mean ± SD of three independent experiments.

Several proteins, such as AUF1, HuR, TTP, etc. have been identified to exert a defined role in regulating mRNA turnover. To address which of these factors regulates FST mRNA expression, we transfected cells with siRNA targeting HuR, AUF1, CP1 or RNP K and examined FST mRNA level. Data showed that down-regulation of AUF1 induced a 2-fold increase of FST mRNA (Supplementary Figure S1). To understand the role of AUF1 in regulation of FST expression, we examined FST protein and mRNA levels after knocking down AUF1. Both protein level and mRNA level (∼2-fold increase) of FST increased after AUF1 siRNA transfection (Figure 3A and B). To dissect which isoform of AUF1 contributes to FST down-regulation, we further expressed each isoform in AUF1-deficient cells. Data showed that each of AUF1 isoforms decreased the expression of FST. The mixture of AUF1 isoforms also significantly decreased the expression of FST (Figure 3A and B). To test whether decrease of FST mRNA by AUF1 is due to a quick mRNA turnover, we detected FST mRNA stability after AUF1 knockdown and rescue. Knockdown of AUF1 increased the half-life of FST mRNA (>8 h versus 6.0 h) while further expression of AUF1 isoforms mixture decreased half-life (5.1 h) (Figure 3C). We also detected the destabilizing effect of AUF1 on FST 3′UTR by using Luc reporter system. Data showed that down-regulation of AUF1 increased luciferase activity of FST 3′UTR construct but the vector only and ARE-deleted construct were not affected (Figure 3D). These data indicated that AUF1 decreased FST mRNA stability through the ARE in 3′UTR.

### AUF1 binds to FST mRNA through ARE and dissociates during glucose deprivation

**Figure 4.**
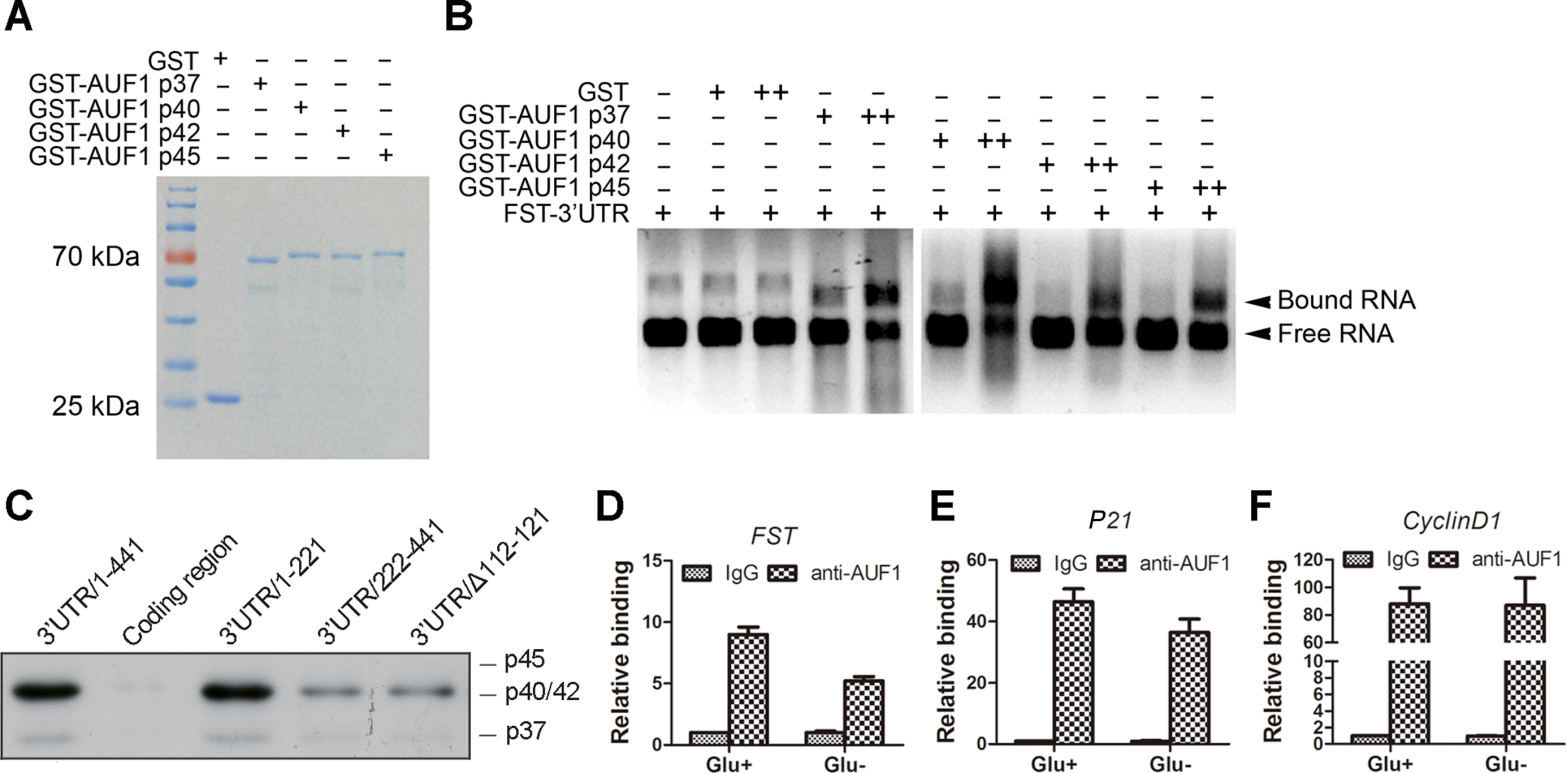
AUF1 binds to FST mRNA and dissociates during glucose deprivation. (**A**) GST and GST-AUF1 isoforms were expressed in *E. coli*, puriﬁed and analysed by Coomassie blue staining. (**B**) FST 3′UTR RNA was incubated with increasing amounts of the indicated proteins. RNA–protein interaction was evaluated by RNA gel shift experiments. (**C**) Pull-down assay was performed using biotinylated FST RNA fragments and HeLa whole-cell lysates followed by immunoblotting. (**D**, **E**, **F**) Immunoprecipitation was performed in cells cultured with or without glucose using anti-AUF1 antibody or normal immunoglobulin G (IgG). FST mRNA (D), p21 mRNA (E) or Cyclin D1 mRNA (F) was determined by RT-qPCR.

To confirm the direct binding between AUF1 and FST 3′UTR, we expressed GST-AUF1 isoforms, p37, p40, p42 and p45 in *E. coli*, purified them (Figure 4A), and analyzed their binding to FST 3′UTR using RNA gel-retardation assays. Data revealed that all the isoforms of AUF1 indeed bind to FST 3′UTR, and the p40 displayed the strongest binding activity (Figure 4B).To identify the potential FST mRNA region(s) recognized by AUF1, we performed *in vitro* RNA pull-down assays. PCR products corresponding to the coding region (CR), the 3′UTR of the FST mRNA or its mutants were transcribed *in vitro*, and the resulting biotinylated RNAs were used in pull-down assays. Data revealed that AUF1 binds to truncated FST 3′UTR containing the first ARE but not the coding region. Deletion of the first ARE significantly reduced AUF1 binding, which is consistent with luciferase assays (Figure 4C). These results indicated that AUF1 binds to ARE in FST 3′UTR directly.

We next asked whether the association between AUF1 and FST mRNA changed during glucose deprivation. Immunoprecipitation reactions were carried out with an anti-AUF1 antibody from control or glucose-deprived cell lysates, and the abundance of FST mRNA in the IP material was subsequently assessed by RT-qPCR. As shown in Figure 4D, glucose deprivation resulted in a marked decrease in the association of AUF1 with FST mRNA. To detect subcellular binding of AUF1 with FST mRNA, we did cytoplasm/nucleus fractionation and performed immunoprecipitation from each fraction. Data showed that the nucleus had less FST mRNA (∼3.5% of cytoplasmic FST mRNA). AUF1 antibody could immunoprecipitate much less FST mRNA from the nucleus than the cytoplasm (0.1% versus 0.9% of total cytoplasmic FST mRNA) (Supplementary Figure S2). These data indicated that AUF1 binds to FST mRNA mainly in the cytoplasm.

To get insight on the specificity of AUF1 function, we also detected the binding between AUF1 and the other two target mRNAs, p21 and Cyclin D1 (27). Data revealed that p21 but not Cyclin D1 mRNA dissociated in response to glucose deprivation (Figure 4E and F). This reduced interactions between AUF1 and its target mRNAs were not due to altered AUF1 IP efficiency because the immunoprecipitated AUF1 protein was the same (Supplementary Figure S3). Our data also showed that the mRNA level of p21 but not Cyclin D1 is increased after glucose deprivation treatment (Supplementary Figure S4). These data suggested that AUF1 might regulate a subset of its target mRNAs expression during glucose deprivation.

### Glucose deprivation promotes cytoplasm to nucleus shuttling of AUF1

**Figure 5.**
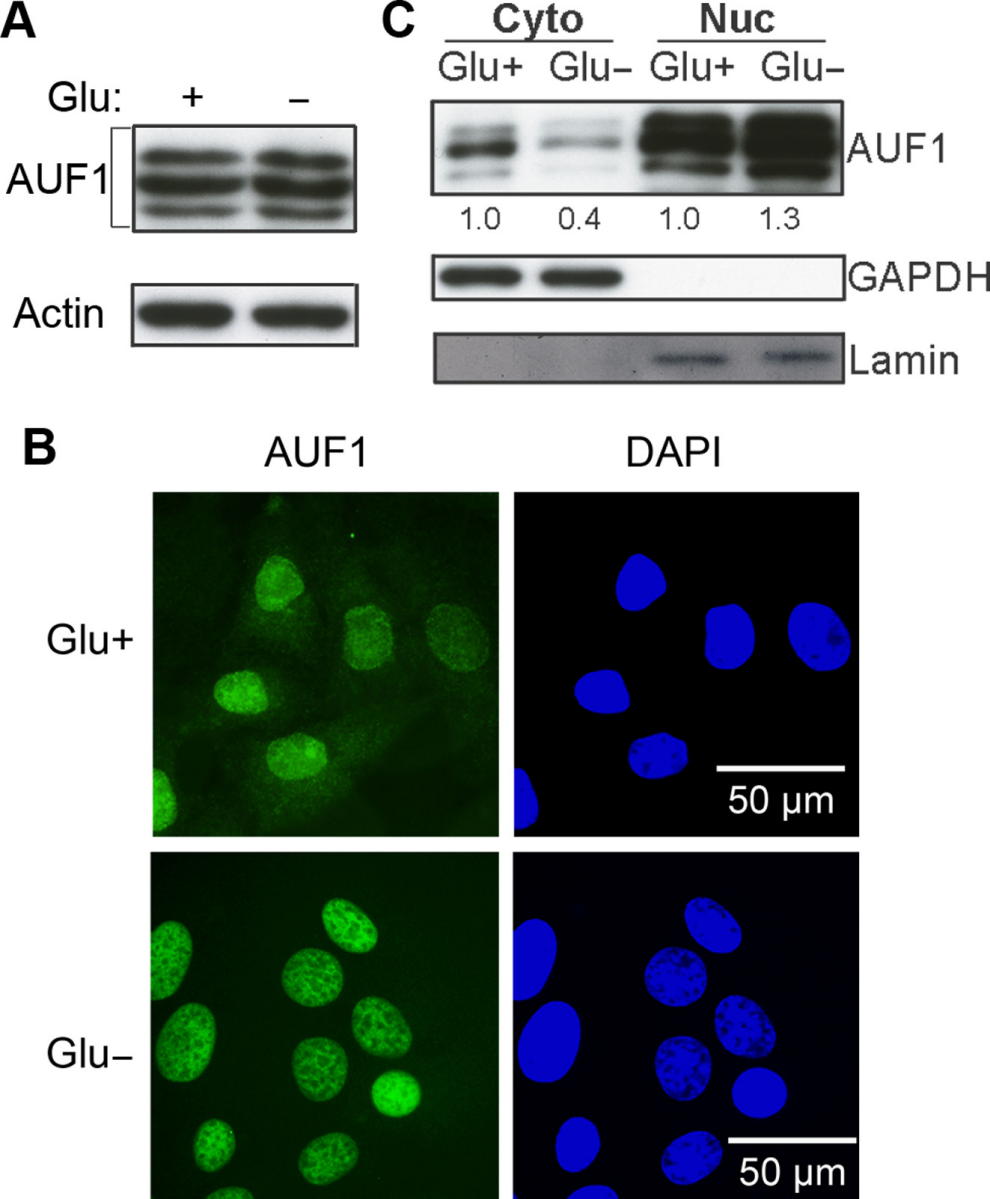
Glucose deprivation promotes cytoplasm to nucleus translocation of AUF1. (**A**) The protein level of AUF1 in HeLa cells incubated with or without glucose for 24 h. (**B**) HeLa cells were incubated with or without glucose for 24 h and then stained with AUF1 monoclonal antibodies. (**C**) AUF1 protein expressions in cytoplasmic (Cyto), and nuclear (Nuc) fractions were analyzed by immunoblotting. Lamin and GAPDH were used as nuclear and cytoplasmic protein marker.

The total protein levels of AUF1 were similar to HeLa cells cultured with or without glucose (Figure 5A), indicating that the change in AUF1/FST mRNA association was not due to the induction of AUF1 expression in glucose-starved cells. We hypothesized that translocation of AUF1 might be involved in the induction of FST mRNA. To test our hypothesis, we cultured HeLa cells with or without glucose for 24 h and examined the cellular localization of AUF1 using immunofluorescence staining. AUF1 was localized in both nucleus and cytoplasm when HeLa cells were cultured with glucose. In contrast, cytosolic AUF1 level was significantly decreased when cells were deprived of glucose (Figure 5B). We further confirmed the reduction of AUF1 in the cytosol by using nuclear/cytoplasmic fractionation approach. Consistently, the level of cytoplasmic AUF1 in HeLa cells cultured without glucose was decreased by ∼60%, compared with cells cultured with glucose (Figure 5C). These observations indicated that glucose deprivation induces translocation of AUF1 from cytoplasm to nucleus in HeLa cells.

### AMPK phosphorylation mediates nuclear translocation of AUF1 and FST induction during glucose deprivation

**Figure 6.**
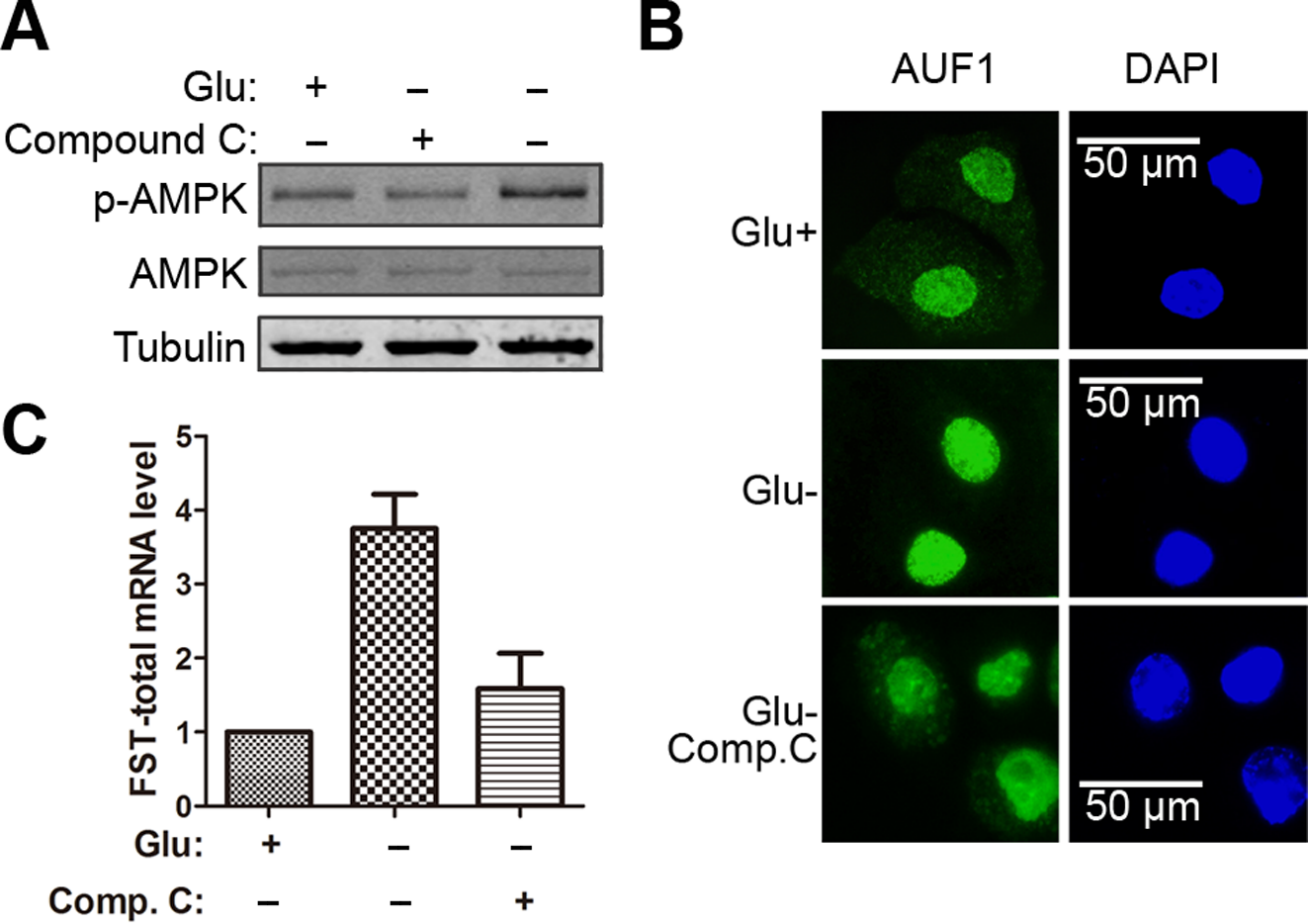
AMPK phosphorylation mediates AUF1 nuclear translocation and FST induction during glucose deprivation. HeLa cells were treated with or without glucose or together with compound C for 12 h. (**A**) Phosphorylation of AMPK was analyzed by immunoblotting. (**B**) The localization of AUF1 was detected by immunofluorescence staining. (**C**) FST mRNA levels were measured with real-time qPCR.

AMP-activated protein kinase (AMPK), a serine–threonine kinase, plays an essential role in cellular energy sensing. This kinase is activated under ATP-depleted conditions such as glucose deprivation by an increase in AMP level (30). To test whether AMPK is involved in glucose deprivation-induced FST expression, we used compound C to inhibit AMPK activation (Figure 6A). As a result, compound C treatment blocked the nuclear translocation of AUF1 (Figure 6B) and the increase of FST mRNA (Figure 6B) in response to glucose deprivation, indicating that AMPK activation mediates AUF1 nuclear translocation and FST induction during glucose deprivation.

### AUF1 confers sensitivity to glucose deprivation-induced apoptosis by suppressing FST expression

**Figure 7.**
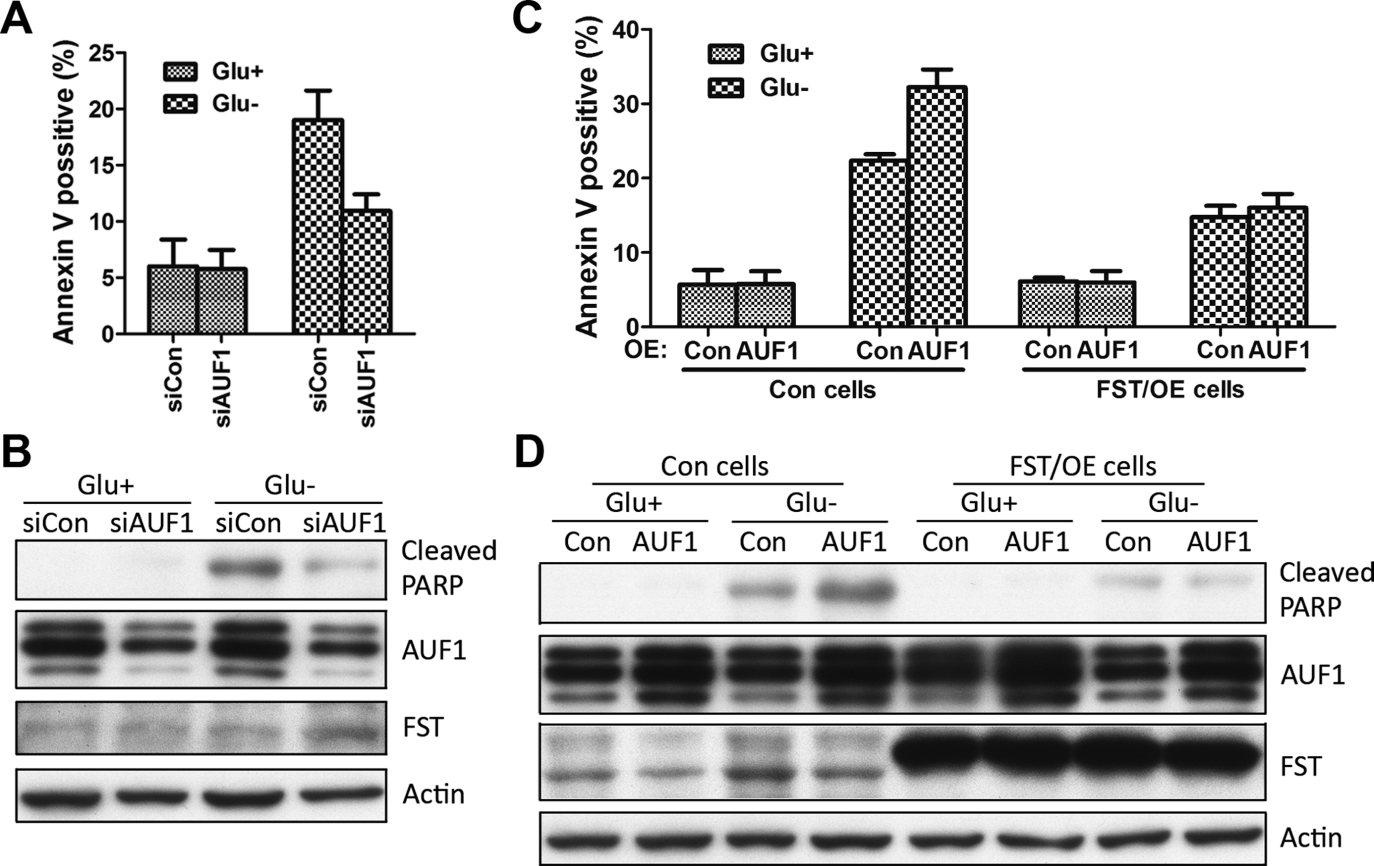
AUF1 confers sensitivity to glucose deprivation-induced apoptosis by FST reduction. (**A**, **B**) HeLa cells transfected with siRNAs targeting AUF1 gene or control siRNAs were grew with or without glucose for 24 h. (A) Apoptotic cells were monitored by annexin V-FITC staining and flow-cytometry analysis. Data shown are mean ± SD of three independent experiments. (B) PARP cleavage was detected by immunoblotting. (**C**, **D**) HeLa cells expressing AUF1, FST, or both were grew with or without glucose for 24 h. (C) Apoptotic cells were monitored by annexin V-FITC staining and flow-cytometry analysis. Data shown are mean ± SD of three independent experiments. (D) PARP cleavage was detected by immunoblotting.

Data described above demonstrated that AUF1 is a negative regulator of FST expression. Our previous studies have shown that FST prevents glucose deprivation-induced apoptosis (13). We therefore sought to determine the function of AUF1 in glucose deprivation-induced apoptosis. HeLa cells transfected with scrambled siRNA or siRNAs targeting AUF1 were cultured with or without glucose for 24 h and cell apoptosis was measured using annexin V staining. The fractions of annexin V-positive cells were low in both groups (5.9 ± 2.4% and 5.8 ± 1.7%, respectively) under normal glucose condition. Glucose deprivation markedly increased cell apoptosis in control cells (19.0 ± 2.6%), and AUF1 elimination reduced the apoptotic tendency to 10.9 ± 1.5% (*P* < 0.01 compared with control) (Figure 7A), indicating an apoptosis-promoting effect of AUF1. The cleavage of PARP is recognized as an apoptosis marker. Our data revealed that PARP was cleaved after glucose deprivation and down-regulation of AUF1 decreased PARP cleavage (Figure 7B).

To further confirm the promoting effect of AUF1 on apoptosis, we overexpressed AUF1 in HeLa cells. As expected, the apoptotic tendency induced by glucose deprivation was significantly increased in AUF1 expressing cells compared to control (32.2 ± 2.4% versus 22.3 ± 0.9%, *P* < 0.01). To investigate whether AUF1 regulates cell apoptosis by down-regulating FST expression, we overexpressed FST. The FST construct did not have the 3′UTR and was resistant to AUF1-mediated degradation. FST expressing cells showed less apoptosis (14.8 ± 1.5%) compared with control, indicating a protective role of FST. Co-expression of AUF1 did not further promote apoptosis in FST expressing cells (16.0 ± 1.9%) (Figure 7C and D), suggesting that AUF1-promoted apoptosis occurs through reduction of FST expression.

## DISCUSSION

**Figure 8.**
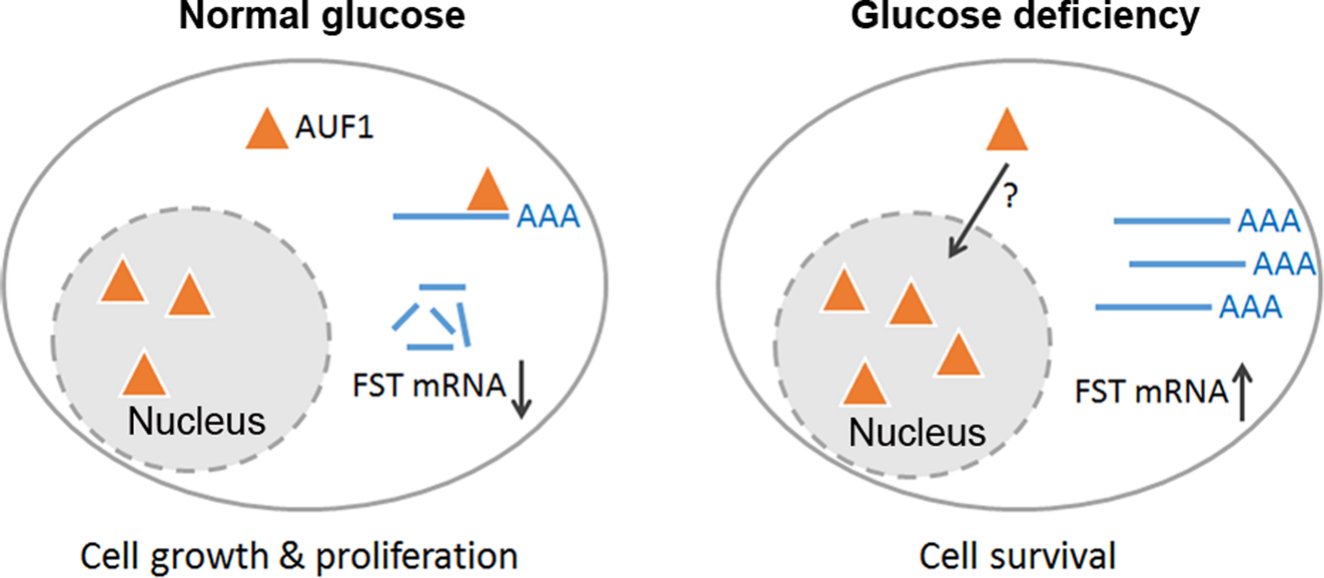
A schematic model for AUF1-promoted FST mRNA decay in regulating cell apoptosis under glucose deprivation. Under normal glucose condition, cytosolic AUF1 binds to FST mRNA and promotes its degradation. Glucose deprivation triggers nuclear translocation of AUF1. Dissociation of AUF1 from the FST mRNA leads to significant increase in FST mRNA stability. Up-regulation of FST promotes cancer cell survival under glucose-deprived condition.

We have previously reported that FST plays an important role in cellular energy homeostasis and its expression level is up-regulated during glucose deprivation (13). However, it is unknown how FST expression is regulated. In this study, we demonstrated that an AU-rich element within the 3′UTR of the FST transcripts mediates the rapid mRNA turnover. RNA–protein interaction and overexpression/knockdown studies revealed that AUF1 binds to FST mRNA directly through ARE and promotes degradation of FST transcripts under normal condition. Glucose deprivation induces dissociation of AUF1 from FST mRNA, and thus increases the expression of FST, which protects cells from glucose deprivation-induced apoptosis. Altogether our data provided direct evidence that AUF1 acts as a negative regulator for FST expression, allowing a quick up-regulation of FST expression during energy deficiency stress (Figure 8).

AREs are sequence elements of 50–150 nt that are rich in adenosine and uridine bases. They are located in the 3′UTRs of mRNAs with a short lifetime, allowing a quick regulation during cellular environmental changes. AREs may promote or reduce mRNA degradation depending on the cellular context and the precise stimulus. Our data showed that ARE in FST mRNA acts as a destabilizing element, as demonstrated in luciferase assays. *In vitro* studies have shown that the nonamer UUAUUUAUU is the minimal AU-rich sequence motif that destabilizes mRNA. Removal of one uridine residue from either end of the nonamer (UUAUUUAU or UAUUUAUU) results in a decrease of potency of the element, while removal of a uridine residue from both ends of the nonamer (UAUUUAU) eliminates detectable destabilizing activity (31). Consistent with this rule, ARE motif identified in FST mRNA is a typical nonamer in sequence. Deletion of the ARE abolished the destabilizing effect of FST 3′UTR and its binding to AUF1, indicating that this ARE is necessary for AUF1-mediated FST mRNA decay (Figure 2). However, one nonamer alone is not enough to promote mRNA degradation, as insertion of a single nonamer in the 3′UTR can only produce a modest effect on the stability of a reporter mRNA (31,32). We found that there is another nonamer in the 3′UTR of FST mRNA that does not have destabilizing role, suggesting that the destabilizing effect of ARE motif depends on its context, such as adjacent sequence and secondary structure. This was observed in the context of the core ARE from TNF-α mRNA, where RNA secondary structure affects AUF1 binding affinity (33).

Our data demonstrated that AUF1 is the *trans*-acting factor that promotes FST mRNA decay. First, silencing of AUF1 increased the half-life of FST mRNA while further expression of AUF1 decreased half-life to the level of control group (Figure 3C). Luciferase assays further consolidated the destabilizing role of AUF1 on 3′UTR of FST mRNA (Figure 3D). Importantly, *in vitro* RNA binding assay and RNA pull-down experiments provided solid evidence that AUF1 binds to FST mRNA directly through ARE (Figure 4A–C). The binding between AUF1 and FST mRNA was regulated by nuclear translocation of AUF1 regulated by extracellular glucose availability (Figure 5). As a matter of fact, subcellular localization change is an important mechanism to regulate AUF1 activities. For instance, anisomycin, an activator of MAPKs, causes shuttling of AUF1 from nucleus to cytoplasm (34). Heat shock induces nuclear and perinuclear localization of AUF1 (35). Our data showed AMPK, the key sensor of cellular energy level, was involved in nuclear translocation of AUF1 during glucose deprivation (Figure 6). However, how AMPK regulates AUF1 shuttling between nucleus and cytoplasm is unknown. Since the total protein level of AUF1 does not change, one may hypothesize that the modifications of AUF1 might change during glucose deprivation. Phosphorylation is an important modification for AUF1. The p40 isoform of AUF1 was reported to be phosphorylated at Ser^83^ and Ser^87^, which affects AUF1 degradation (36,37) or its affinity for the ARE (38). However, currently we do not know whether it also affects protein localization. Further studies on the phosphorylation of AUF1, including the specific kinases and the potential phosphorylation sites would illustrate the mechanism regulating AUF1 localization.

The effect of AUF1 on the establishment or progression of cancer is not clear. Some reports showed that AUF1 has an anti-tumorigenic function. The protein destabilizes mRNAs encoding the anti-apoptotic protein Bcl-2 (39) and the proliferative protein Cyclin D1 (40). It can also suppress the expression of pro-inflammatory factors (e.g. IL-6, GM-CSF, iNOS, COX-2) by promoting decay of the relative coding mRNAs, and thereby suppressing a pro-transformation state, which is important for tumor progression (41). We demonstrated that AUF1-promoted cell apoptosis during glucose deprivation. Since the increased resistance of cancer cells to glucose deficiency contributes positively to tumor progression (42), out data suggested that AUF1 might inhibit solid tumor development via destabilization of FST mRNA. However, there is also evidence that support a pro-tumorigenic role of AUF1. Genetic overexpression of p37 isoform of AUF1 in mice led to the development of sarcomas in different tissues (43). Since different isoforms of this protein have different subcellular distributions and might have distinct functions (44), it is unclear whether the other three isoforms also lead to tumor formation. The inconsistencies of AUF1 studies in diverse model systems suggest that the function of AUF1 is regulated by many parameters including AUF1 dosage, isoform selectivity, and the availability of other protein co-factors. Therefore, AUF1 might play different roles in different types of cancers and in different stages during tumor progression.

In summary, we demonstrated that glucose deprivation induces translocation of AUF1 from cytoplasm to nucleus which allows dissociation of AUF1 from FST mRNA and perhaps other mRNAs. This leads to stabilization of FST mRNA and promotes cell survival. Investigating how the AUF1–FST pathway regulates cancer cell energy homeostasis using mouse models will provide insights into the mechanism of solid tumor development and may lead to potential intervention strategies for cancer treatment.

## SUPPLEMENTARY DATA

Supplementary Data are available at NAR Online.

SUPPLEMENTARY DATA
