# Supplementary material for: Reduction of AUF1-mediated follistatin mRNA decay during glucose starvation protects cells from apoptosis
Source: Nucleic Acids Res. 2014 Aug 26;42(16):10720–30. doi: 10.1093/nar/gku778 (PMC4176339; doi:10.1093/nar/gku778)
Supplement: SUPPLEMENTARY DATA [file supp_42_16_10720__index.html]

Reduction of AUF1-mediated follistatin mRNA decay during glucose starvation protects cells from apoptosis — Reduction of AUF1-mediated follistatin mRNA decay during glucose starvation protects cells from apoptosis — SUPPLEMENTARY DATA 

# Reduction of AUF1-mediated follistatin mRNA decay during glucose starvation protects cells from apoptosis

## SUPPLEMENTARY DATA

**Files in this Data Supplement:**

- SUPPLEMENTARY DATA
