# Supplementary material for: Reduction of AUF1-mediated follistatin mRNA decay during glucose starvation protects cells from apoptosis
Source: Nucleic Acids Res. 2014 Aug 26;42(16):10720–30. doi: 10.1093/nar/gku778 (PMC4176339; doi:10.1093/nar/gku778)
Supplement: SUPPLEMENTARY DATA [file supp_gku778_nar-00775-a-2014-File010.pdf]

Supplementary information

Supplementary Tables

Table S1. Primer design for generation of FST 3'UTR mutants.

UTR mutant	Primer set
pGL3-UTR/1-441	UTR-1-for-luc + UTR-441-rev
pGL3-UTR/1-221	UTR-1-for-luc + UTR-221-rev
pGL3-UTR/222-441	UTR-222-for-luc + UTR-441-rev
pGL3-UTR/Δ112-121=UTR/1-111	UTR-1-for-luc + UTR-111-rev
+ UTR/122-441	UTR-121-for + UTR-441-rev
pcDNA-UTR/1-441	UTR-1-for-cdna + UTR-441-rev
pcDNA-UTR/1-221	UTR-1-for-cdna + UTR-221-rev
pcDNA-UTR/222-441	UTR-222-for-cdna + UTR-441-rev
pcDNA-UTR/Δ112-121=UTR/1-111	UTR-1-for-cdna + UTR-111-rev
+ UTR/122-441	UTR-121-for + UTR-441-rev

Table S2. Sequence of primers used for plasmid construction.

Primer Name	Sequence
UTR-1-for-luc	GC<u>TCTAGA</u>ACTCTCTATAAGTGTTC
UTR-1-for-cdna	CCC<u>AAGCTT</u>ACTCTCTATAAGTGTTC
UTR-441-rev	GC<u>TCTAGA</u>GTGAGTGAATAGTAAGG
UTR-221-rev	GC<u>TCTAGA</u>GTAGACCTCACAATG
UTR-222-for-luc	GC<u>TCTAGA</u>TGGATGAGGCCCATAGT
UTR-222-for-cdna	CCC<u>AAGCTT</u>TGGATGAGGCCCATAGT
UTR-111-rev	G<u>GAATTC</u>GCATACACTTATTTAC
UTR-121-for	G<u>GAATTC</u>GGGGGGAAAACTATAC

Supplementary Figures

Fig. S1.

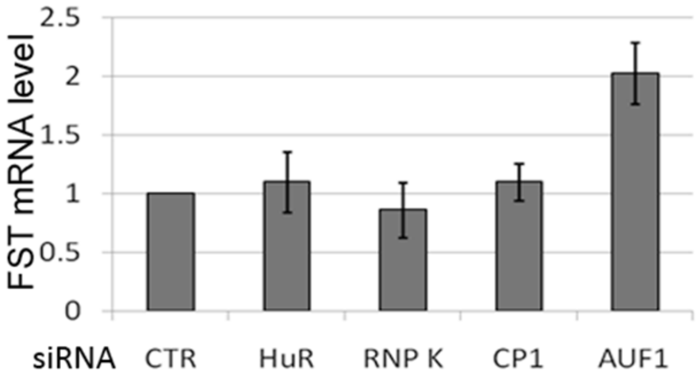

HeLa cells were transfected with control siRNA (CTR) or siRNA targeting HuR, RNP K, CP1, AUF1. The mRNA level of FST were detected.

Fig. S2

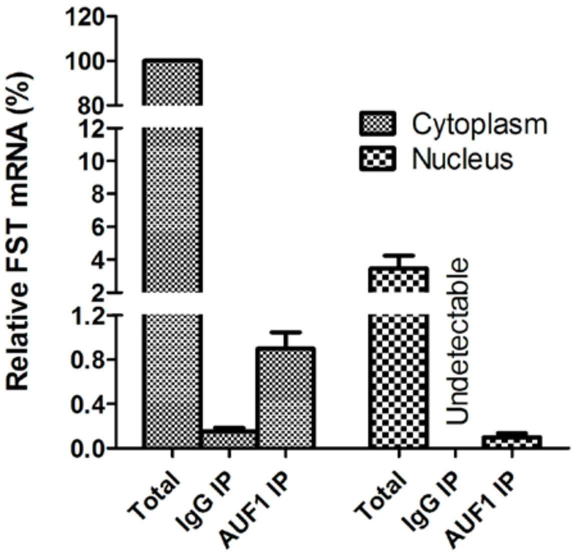

Immunoprecipitation was performed from cytoplasmic and nuclear fractions using anti-AUF1 antibody or normal immunoglobulin G (IgG). FST mRNA levels in input (10% of total fraction), IgG IP group and AUF1 IP group were detected by real-time qPCR. Total cytoplasmic FST mRNA level (10 fold of cytoplasmic input) was set to 100.

Fig. S3

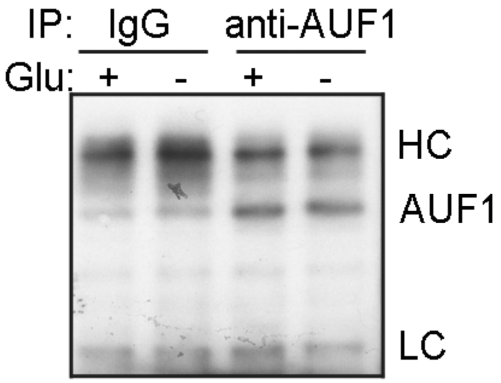

Immunoprecipitation was performed in cells cultured with or without glucose using anti-AUF1 antibody or normal immunoglobulin G (IgG), and immunoprecipitation efficiency was detected by immunoblotting.

Fig. S4

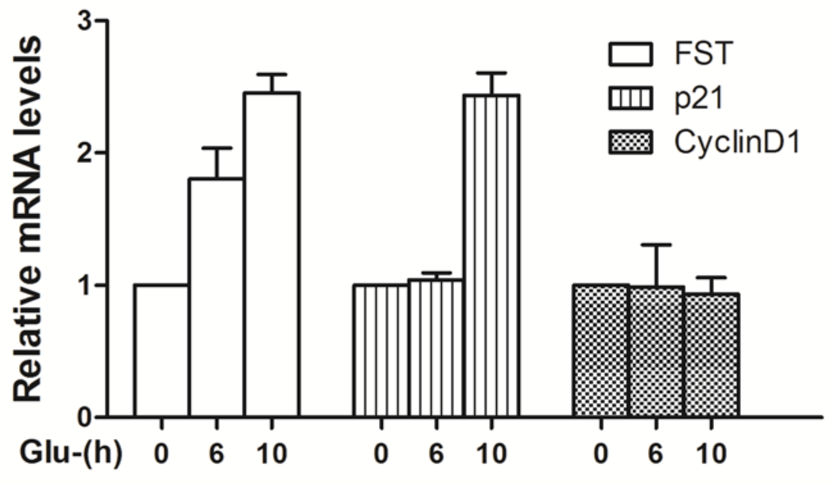

HeLa cells were incubated with or without glucose for indicated time and the mRNA levels of FST, p21 and Cyclin D1 were measured with real-time qPCR.
